# Sentinel Node Biopsy in Ductal Carcinoma in Situ: Is it Justifiable?

**DOI:** 10.7759/cureus.13062

**Published:** 2021-02-01

**Authors:** Nour Al-Shurbasi, Natalie A Hirst, Stanley Kohlhardt

**Affiliations:** 1 Breast Surgery, Chesterfield Royal Hospital, Chesterfield, GBR; 2 Breast Surgery, Sheffield Teaching Hospitals NHS Foundation Trust, Sheffield, GBR

**Keywords:** alnd: - axillary lymph node dissection, axillary clearance, micro metastases, ductal carcinoma in situ, sentinel node biopsy

## Abstract

Background: For invasive breast cancer, sentinel node biopsy (SNB) is an acceptable alternative to axillary node clearance (ANC), although in the recent era, its role is under review. In ductal carcinoma in situ (DCIS), the benefit of SNB is even less well defined. Despite this, guidelines still recommend that it is performed in selected cases of DCIS. The aim of our study was to evaluate the diagnostic value of performing SNB in DCIS.

Methods: Patients with a diagnosis of DCIS who underwent axillary staging with SNB between 2008-2019 in our large volume tertiary centre were identified and included in the study.

Results: Out of the 48 patients who were identified, four patients had a positive SNB (8%). Two of those patients were found to have micro metastatic disease. None of the patients with a positive SNB had local or systemic recurrence (median follow up: 40 months). One non-breast cancer-related mortality was reported. Two patients were identified who had recurrent disease, one with an invasive recurrence in the breast, and the other with systemic recurrence in the form of bone disease. Both of these patients had a negative SNB.

Conclusion: Our results confirm that performing axillary staging with SNB in DCIS is not justifiable, as it does not affect patient outcomes. This supports the emerging evidence that being more surgically conservative may decrease morbidity without affecting patient survival.

## Introduction

Ductal carcinoma in situ (DCIS) is defined as a type of breast cancer in which there is an abnormal proliferation of cells within the milk ducts which have not invaded beyond the basement membrane to the surrounding tissues. It is, therefore “in situ” cancer, which does not spread to the lymph nodes or distant organs. DCIS accounts for approximately 20% of screen-detected breast tumors in the UK [1]. The vast majority of cases are diagnosed on screening, with up to 90% of cases being impalpable and asymptomatic. Only 10% are associated with symptoms which include a mass, nipple discharge, and ulceration (Paget’s disease). The incidence of DCIS has increased in recent years, widely attributed to the use of screening programs as mentioned, but also better technological advancements improving diagnosis. Over 60,000 women are diagnosed with DCIS each year in the USA, over 7000 in the UK, and over 2500 in the Netherlands [2].

Due to the characteristic inability to spread, a biopsy to assess metastasis to the lymph nodes in the axilla in DCIS would logically not be considered necessary. However, in approximately 20%-30% of resections for DCIS, invasive cancer is discovered in the post-operative histological specimen. It is well established that invasive cancer correlates with sentinel node metastasis, with an incidence of involved nodes of 15.6% compared with 2% in pure DCIS [3]. It is not possible to pre-operatively predict which patients with DCIS will also have occult invasive disease. Parameters which are considered to convey an increased risk are: size >50 mm, presence of extensive calcifications on mammography, and clinical presentation with a palpable lump. Sentinel node biopsy (SNB) is therefore currently offered to these patients, with this justification. In planned mastectomy for widespread DCIS in the breast, SNB is also commonly performed as it would not be feasible at a later stage should an invasive component subsequently be found [4]. Despite these criteria, SNB is still reported to be performed in surgery for DCIS in up to 51% of cases [3].

SNB is carried out by a radioisotope +/- blue dye subcutaneous injection into the breast, in order to trace the lymphatic channels to the first draining lymph node of the breast. The procedure involves minimal dissection and division of lymphatic channels compared to formal axillary node clearance (ANC); nevertheless, complications are extensive and confer significant morbidity. They include infection, seroma, hematoma, anaphylaxis, axillary vein injury, shoulder stiffness, limitation in shoulder range of motion, and lymphedema [5].

Recent focus with the advent of adjuvant therapies such as radiotherapy has been in tailoring breast cancer management strategies to the individual, with the aim of maximizing the benefits and avoiding unnecessary risks. In known invasive breast cancer, there is a shift towards minimizing invasive dissection in the axilla in clinically node-negative patients with one to two sentinel node metastases with seemingly no adverse effect on mortality [2]. This in turn has led to trials investigating whether SNB can be omitted altogether in breast-conserving surgery under select conditions [6]. If invasive axillary treatment can be safely minimized or avoided in invasive breast cancer, this prompts discussion for re-evaluating axillary management in DCIS. However, evidence for this is currently inadequate due to the paucity of data in the literature and a lack of long-term follow-up studies [7].

The aim of this study is to evaluate whether SNB is a justifiable management strategy for DCIS, or whether it can be safely omitted.

## Materials and methods

All consecutive patients diagnosed with DCIS in our large volume tertiary center from 2008 to 2019 were retrieved from two databases: the local breast reconstruction database and the coding department database. Fifty-one patients were identified who fulfilled our detailed search criteria, namely patients who had a diagnosis of DCIS on core biopsy and subsequently had axillary surgery performed, three patients were excluded as their DCIS was recurrent, with a previous diagnosis of ipsilateral carcinoma in the past. The nature of the axillary surgery was either as an upfront SNB, intra operatively, or post operatively as completion axillary staging when invasive disease was found on the resection specimen. Clinically node-negative patients were included. Patients with ipsilateral invasive breast cancer were excluded.

Data were extracted electronically for all patients and included basic patient demographics such as age, core needle biopsy methods, surgical procedures, and pathology reporting on tumor type, grade, presence of microinvasion, size, and (sentinel) lymph nodes.

In our pathology department, specimens are processed according to national guidelines (National Institute for Health and Clinical Excellence (NICE)). In accordance with this, whenever there is uncertainty in histopathological core biopsy specimens, an image-guided vacuum-assisted biopsy is performed. In the event of high clinical suspicion in patients presenting with a palpable lump or thickening, a clinical core (non-image guided) biopsy is performed, or patients undergo diagnostic excisional biopsy.

Statistical analysis of data was carried out using Statistical Package for the Social Sciences (SPSS Inc., Chicago, IL). Correlations between variables which are considered to be predictors of the risk of synchronous invasive disease in patients with DCIS were analyzed using Pearson’s coefficient of correlation.

## Results

We identified a total of 51 patients who had DCIS on their core biopsies and subsequently underwent an SNB. Three patients were excluded, due to their DCIS being a local recurrence.

Upfront (prior to cancer surgery) SNB was performed in 2/48 (4%) cases, in both cases the decision to stage the axilla upfront was taken due to the presence of large areas of calcifications in young patients. Surgery for DCIS and simultaneous SNB was carried out in 41/48 (85%) of patients. In 3/48 (6%) of cases, axillary staging was performed after the initial cancer resection surgery, as invasive cancer was discovered in the histological specimen. 2/48 (4%) patients had mastectomies for DCIS without any staging of the axilla. Out of these 48 patients, 4/48 (8%) had a positive SNB; 2/48 (4%) patients had micro metastases which were detected intra-operatively using one-step nucleic acid amplification (OSNA), and 2/48 (4%) had macro metastases.

None of the patients with positive sentinel nodes had local or systemic recurrence, median follow up 40 months, a minimum of two months, and a maximum of 10 years and 11 months (confidence level at 95%=1). Those patients who had positive sentinel nodes were analyzed case by case to identify factors which might potentially be associated with sentinel node positivity (Table 1).

**Table 1:** Risk factors in patients with DCIS and positive sentinel nodes in our cohort DCIS: ductal carcinoma in situ

	Patient A	Patient B	Patient C	Patient D
DCIS Size (mm)	53	35	60	130
Nuclear Grade	High Nuclear Grade (HNG)	High Nuclear Grade (HNG)	High Nuclear Grade (HNG)	Low Nuclear Grade (LNG)
Microinvasion	Present	Not present	Not present	Not present
Sentinel nodes	Three macro metastases	one micro metastasis	one macro and one micro metastasis	1 micro metastasis
Age at diagnosis (years)	34	76	67	73
Local recurrence	None	None	None	None
Systemic recurrence	None	None	None	None

An ANC was performed in one patient (Patient A) who had three macro metastases in her SNB. No invasive disease was found in her breast despite re-examination of her breast specimen by a further two consultant histopathologists. Her sentinel nodes demonstrated metastatic high-grade carcinoma and expression of human epidermal growth factor receptor 2 (HER-2), therefore, she was commenced on chemotherapy and Herceptin. She underwent an ANC following her adjuvant treatment which did not demonstrate any further involved nodes. Both Patients B and D, who had micrometastasis to one node only never had any further axillary surgery, in line with the NICE guidance. Patient C who had one micrometastatic node along with one macrometastatic node, never had a completion ANC, as they were too frail for the procedure.

We considered risk factors in the literature which are known to increase the likelihood of having invasive disease in patients who have DCIS. Micro invasion was associated with a higher risk of sentinel node positivity, Pearson’s correlation -.425-(Sig (2 tailed)) .003 (correlation is significant at the 0.01 level (2 tailed). No other factors in our cohort demonstrated a significant correlation between DCIS and having a positive sentinel node including the size and grade of DCIS, and the age of the patient at diagnosis (Table 2).

**Table 2:** Pearson's correlation of potential risk factors for positive sentinel node biopsy in DCIS patients * Correlation is significant at the 0.01 level (2-tailed). DCIS: ductal carcinoma in situ; SNB: sentinel node biopsy

		age at diagnosis	DCIS Grade	microinvasion	DCIS size	SNB Result
age at diagnosis	Pearson Correlation	1	-.085-	.100	-.128-	-.015-
	Sig. (2-tailed)		.547	.500	.367	.919
	N	52	52	48	52	50
DCIS Grade	Pearson Correlation	-.085-	1	.086	.080	-.048-
	Sig. (2-tailed)	.547		.562	.572	.739
	N	52	52	48	52	50
microinvasion	Pearson Correlation	.100	.086	1	.104	-.425-^*^
	Sig. (2-tailed)	.500	.562		.483	.003
	N	48	48	48	48	46
DCIS size	Pearson Correlation	-.128-	.080	.104	1	.162
	Sig. (2-tailed)	.367	.572	.483		.260
	N	52	52	48	52	50
SNB Result	Pearson Correlation	-.015-	-.048-	-.425-^*^	.162	1
	Sig. (2-tailed)	.919	.739	.003	.260	
	N	50	50	46	50	50

In our cohort of patients diagnosed with DCIS, two patients were identified who had recurrent disease. One patient had an invasive recurrence in the breast, and the other patient had systemic recurrence in the form of bone disease. Both of these patients had a negative SNB.

We found that the survival of patients was not affected by a diagnosis of DCIS, nor by having involved sentinel nodes, regardless of whether these were micro or macro metastasis. The only mortality recorded was non-breast cancer related, and was linked to other comorbidities of the patient.

## Discussion

With the widespread dissemination of breast cancer screening in the Western World, breast cancer is being diagnosed increasingly frequently. Currently, DCIS accounts for 10%-20% of newly diagnosed breast cancers [8]. 

The influence of the presence of microinvasive disease on outcomes has been hotly debated. Cavaliere et al. [9] suggested that the natural history of microinvasive DCIS resembles DCIS and is different from T1a invasive ductal cancers. They demonstrated no axillary recurrence or distant metastases in their cohort of 31 patients over long-term follow up (mean 75 months). This appears to be in agreement with our findings, where microinvasive disease was correlated with a higher risk of a positive sentinel node but did not affect the survival of patients or confer an increased risk of local recurrence.

We found 8% (4/48) of patients with DCIS also had invasive disease in their operative specimen and went on to have axillary staging. None of these patients subsequently had macro or micro metastases in their sentinel nodes. These findings appear to be in agreement with the conclusions of Fallowfield et al. [10] which suggested that watchful waiting, in comparison to performing axillary lymph node staging, showed no significant differences in regional recurrence rates after a mean follow-up of 6.3 years.

Leading international societies such as the National Comprehensive Cancer Network (NCCN) and the American Society of Clinical Oncology (ASCO), do not recommend axillary lymph node evaluation for patients undergoing BCS for DCIS, as non-invasive breast cancer cannot lead to lymph node metastasis. An SNB may however be performed in patients undergoing total mastectomy, as well as in cases of surgery performed in anatomical sites which would potentially compromise a future procedure (central breast, upper outer quadrant, or axillary tail) and in cases of large volume DCIS [11]. Our results concur that performing an SNB did not affect the oncological outcomes of almost all of our patients. The morbidity of SNB such as lymphoedema is approximately 7.5%, as well as giving rise to other known complications (shoulder stiffness, numbness, infection, etc) [8,12].

A retrospective review conducted in cases of DCIS treated with BCS in the Netherlands [13] between 1998 and 2011 demonstrated a rate of axillary surgical procedures of 43.9% (including 4.5% for ANC). Clearly, this is higher than the guidelines would suggest. Our results are in line with previous research which has shown that axillary staging in DCIS, even if selective, has no correlation with patients’ management plans or outcomes.

One of the limitations of our study was the retrospective design. Although allowing a higher number of study subjects, retrospective cohort studies are known for their selection bias of the studied population. In our study, we tried to obviate this by retrieval of data from two different sources. Another limitation is the relatively small sample size, although this is representative of the small number of axillary staging procedures performed in cases of DCIS.

## Conclusions

Despite the low association between the high-risk characteristics of DCIS and the involvement of axillary nodes, axillary staging via SNB is still routinely performed in selected patients in the UK. In accordance with our findings, SNB in patients with DCIS should not be performed as it is invasive and may confer significant morbidity, and does not improve patients’ oncological outcomes. We are in need of a larger-scale study with robust data analyses to incorporate these findings in the management of all cases of DCIS.

## References

[1] Harvey J, Down S, Bright Thomas R, Winstanley J, Bishop H (2020). Breast Disease Management: A Multidisciplinary Manual. https://oxfordmedicine.com/view/10.1093/med/9780199215065.001.0001/med-9780199215065.

[2] Giuliano AE, Ballman KV, McCall L (2017). Effect of axillary dissection vs no axillary dissection on 10-year overall survival among women with invasive breast cancer and sentinel node metastasis: the ACOSOG Z0011 (Alliance) randomized clinical trial. JAMA.

[3] van Roozendaal LM, Goorts B, Klinkert M (2016). Sentinel lymph node biopsy can be omitted in DCIS patients treated with breast conserving therapy. Breast Cancer Res Treat.

[4] (2021). Early and locally advanced breast cancer: diagnosis and management: NICE guideline [NG101]. https://www.nice.org.uk/guidance/ng101.

[5] Wilke LG, McCall LM, Posther KE (2006). Surgical complications associated with sentinel lymph node biopsy: results from a prospective international cooperative group trial. Ann Surg Oncol.

[6] Gentilini O, Veronesi U (2012). Abandoning sentinel lymph node biopsy in early breast cancer? A new trial in progress at the European Institute of Oncology of Milan (SOUND: Sentinel node vs Observation after axillary UltraSouND). Breast.

[7] Magnoni F, Massari G, Santomauro G (2019). Sentinel lymph node biopsy in microinvasive ductal carcinoma in situ. Br J Surg.

[8] Lyman GH, Temin S, Edge SB (2014). Sentinel lymph node biopsy for patients with early-stage breast cancer: American Society of Clinical Oncology clinical practice guideline update. J Clin Oncol.

[9] Cavaliere A, Scheibel M, Bellezza G (2006). Ductal carcinoma in situ with microinvasion: clinicopathologic study and biopathologic profile. Pathol Res Pract.

[10] Fallowfield L, Francis A (2016). Overtreatment of low-grade ductal carcinoma in situ. JAMA Oncol.

[11] Daly MB, Pilarski R, Berry M (2017). NCCN guidelines insights: genetic/familial high-risk assessment: breast and ovarian, version 2.2017. J Natl Compr Canc Netw.

[12] Lorek A, Stojčev Z, Zarębski W, Kowalczyk M, Szyluk K (2019). Analysis of postoperative complications after 303 sentinel lymph node identification procedures using the sentimag® method in breast cancer patients. Med Sci Monit.

[13] Mitchell KB, Lin H, Shen Y (2017). DCIS and axillary nodal evaluation: compliance with national guidelines. BMC Surg.

